# The role of resveratrol in delivering antioxidant, anti-inflammatory, and anti-apoptotic defense against nephrotoxicity generated by titanium dioxide

**DOI:** 10.1007/s00210-025-03885-7

**Published:** 2025-02-24

**Authors:** Feyza Basak, Tansu Kusat, Tahir Kahraman, Yusuf Ersan

**Affiliations:** 1 Department of Histology and Embryology, Faculty of Medicine, Karabuk University, Karabuk, Turkey; 2 Department of Medical Biochemistry, Faculty of Medicine, Karabuk University, Karabuk, Turkey

**Keywords:** Kidney, Oxidative stress, Rat, Resveratrol, Titanium dioxide

## Abstract

Titanium dioxide is a prevalent food ingredient for human ingestion. We investigated the nephrotoxic effects of titanium dioxide in Wistar albino rats subjected to oral exposure for 14 days. The rats were categorized into four groups (*n* = 8): (1) control (saline solution), (2) exposure to titanium dioxide (30 mg/kg), (3) exposure to resveratrol (100 mg/kg), and (4) exposure to both titanium dioxide and resveratrol. The investigations revealed that the administration of titanium dioxide resulted in considerable histological abnormalities and a significant prevalence of apoptotic cells marked by caspase-3 in the titanium dioxide group, with a markedly elevated quantity and strong staining of cells reacting with 4-HN across the tissue in the kidney. Blood serum assessments revealed that BUN and creatinine levels were elevated in the titanium group relative to the other three groups, with a reduction in these levels observed in the group receiving both titanium and resveratrol (*P* < 0.05). The assessment of oxidative stress markers in kidney tissue revealed that GSH-Px and SOD activity considerably decreased in the titanium dioxide group relative to the other experimental groups. In contrast, MDA levels increased markedly (*P* < 0.05). The activities of GSH-Px and SOD were significantly elevated in the group receiving both titanium dioxide and resveratrol compared to the titanium dioxide-only group (*P* < 0.05). The analysis of inflammation markers TNF-α and IL-6 revealed a substantial rise in their levels in the titanium dioxide group compared to the other groups (*P* < 0.05).

## Introduction

Titanium dioxide (TiO_2_, E171) serves as a whitening agent in several medicinal supplements, medications, confections, chewing gum, sauces, and dairy products including milk and cream, and coffee whiteners (Kirkland et al. 2022). The growing utilization of TiO_2_ in consumer and industrial products prompts apprehensions over its possible health hazards to humans, animals, and the environment (Ayorinde and Sayes 2023). Research indicates the adverse impacts of TiO_2_ on human health (Ayorinde and Sayes 2023). Toxicity is the consequence of TiO_2_ accumulation in various organs, such as the spleen, kidneys, liver, and specific regions of the intestines (Valentini et al. 2019; Brand et al. 2020; Peters et al. 2020; Elgrabli et al. 2015; Geraets et al. 2014). TiO_2_ has been prohibited in the European Union due to its classification as genotoxic by the European Food Safety Authority (Møller and Roursgaard 2024). Currently, there is a lack of data regarding the remediation of organ damage induced by TiO_2_ introduced into the body.

The kidney is regarded as one of the most susceptible organs to toxic agents in the body owing to its elevated blood flow and capacity to concentrate waste materials (L'azou et al. 2002). Recent findings indicate that TiO_2_ buildup is greater in the kidneys than the other organs (Brand et al. 2020; Peters et al. 2020). TiO_2_ has been observed to compromise renal function and induce oxidative stress and inflammation in the kidneys by blocking various antioxidants and detoxifying agents (Gui et al. 2013). Oxidative stress is characterized as a state arising from the disparity between the generation of free radicals and reactive oxygen species and the antioxidant defense mechanisms. Oxidative stress has been documented to adversely affect macromolecules, such as DNA, proteins, and lipids (Chandra et al. 2015).

4-Hydroxynonenal (4-HN) accumulates in elevated concentrations during oxidative stress due to enhanced lipid peroxidation. It possesses cytotoxic, hepatotoxic, mutagenic, and genotoxic characteristics. 4-HN has higher levels in plasma and tissues under circumstances of oxidative stress (Esterbauer and Cheeseman 1990). Oxidative damage is known to promote apoptosis in organisms. Caspase-3 belongs to the effector caspase class and its activation induces apoptosis (Coşkun and Özgür 2011). Titanium dioxide molecules have been shown to induce apoptosis in human embryonic kidney cells (Meena et al. 2012). Recent research indicates that TiO_2_ is more prone to induce excessive generation of reactive oxygen species (ROS) in impacted tissues (Meena et al. 2012). Reports indicate that elevated oxidative damage in the kidney due to titanium dioxide leads to the buildup of proteinaceous substances in the distal tubule lumen and the medullary collecting duct (Meena et al. 2012; Yang et al. 2021).

Recent research has demonstrated the therapeutic advantages of medicinal plants in mitigating chemical toxicity (Riaz et al. 2023). Resveratrol is a phytoalexin produced by plants to protect against environmental stressors (Chang et al. 2011). Resveratrol is linked to numerous beneficial health effects, including anti-inflammatory, anti-cancer, antiviral, anti-aging, antidiabetic, and antihyperlipidemic qualities. Research indicates that resveratrol mitigates oxidative DNA damage more efficiently than alternative antioxidant compounds (Shakibaei et al. 2009; Kawada et al. 1998). Additionally, resveratrol is recognized as an efficient scavenger of reactive oxygen species (Kitada and Koya 2013). Besides scavenging reactive oxygen species (ROS), exogenously administered resveratrol modulates the expression and activity of antioxidant enzymes, such as superoxide dismutase (SOD), glutathione peroxidase (GPx), and catalase (CAT), via enzymatic modification or transcriptional regulation (Robb et al. 2008; Kitada et al. 2011).

This study examined the therapeutic benefits of resveratrol on nephrotoxicity induced by TiO_2_ exposure. We employed histopathological examination alongside anti-caspase-3 and anti-4-HN immunohistochemical antibodies to identify apoptosis and oxidative damage, respectively. We assessed the concentrations of malondialdehyde (MDA) as a terminal result of lipid peroxidation. The activity of kidney superoxide dismutase (SOD) and levels of glutathione peroxidase (GSH-Px) were assessed, along with blood levels of BUN and creatinine. We evaluated the concentrations of proinflammatory cytokines, specifically interleukin-6 (IL-6) and tumor necrosis factor-alpha (TNF-α), in kidney specimens. Furthermore, histological alterations identified in the kidney were assessed using histometric measures.

## Materials and methods

### Drugs and chemicals

#### Titanium dioxide

Titanium dioxide was produced by Alfasol, T2944_f3092, Turkey.

Titanium dioxide powder was suspended in distilled water, and that suspension was vortexed for 10 min before the treatment.

#### Resveratrol

Resveratrol was purchased from Sigma-Aldrich, St. Louis, Germany, 1,185,247–70-4.

### Ethics committee approval

The requisite ethical approval for the investigation was secured by the Karabük University Animal Experiments Local Ethics Committee (2023/2/5).

All the authors indicate that all animal experiments comply with the ARRIVE guidelines and are carried out following the UK Animals (Scientific Procedures) Act, 1986 and associated guidelines, EU Directive 2010/63/EU for animal experiments, or the National Institutes of Health guide for the care and use of laboratory animals (NIH Publications No. 8023, revised 1978).

### Experimental animals

Thirty-two male Wistar Albino rats, weighing between 200 and 240 g, were randomly recruited from the Karabük University Experimental Animal Application Center (DETUM). The individuals were maintained on a 12-h light and 12-h dark cycle in an environment with a temperature of 22 °C and a relative humidity of 50–55%. The subjects, housed in metal cages, were provided with conventional rat pellet feed and had continuous access to food and water.

### Establishment of experimental cohorts and administration of gavage

The chosen rats were randomly categorized as follows.

- Group 1—control group (C): This group received 1 ml of distilled water via gavage for a duration of 14 days (*n* = 8).
- Group 2—TiO_2_ group (T): The subjects in this group received 100 mg/kg (Papp et al. 2020) TiO_2_ (Alfasol, T2944_f3092, Turkey) diluted in 1 ml of distilled water via gavage daily for 14 days (*n* = 8).
- Group 3—resveratrol group (R): The subjects in this group received 30 mg/kg (Kong et al. 2020) resveratrol (Sigma-Aldrich, St. Louis, MO, 1,185,247–70-4) administered daily via gavage in 1 ml of distilled water for 14 consecutive days (*n* = 8).
- Group 4—TiO_2_ and resveratrol group (T + R): The identical treatment administered to the T group was conducted for 14 days, followed by the application of the R group 1 h later (*n* = 8). The rats, categorized into groups, were weighed on a precision scale, and their life weights (BW) were documented.

### Collecting blood and tissue samples

On the 14th day, the rats were euthanized via cervical dislocation following the measurement of their live weights using a precision scale under ketamine-xylazine anesthesia (50 mg/kg–5 mg/kg, respectively) (Struck et al. 2011), and their kidney tissues were collected. One-half of each kidney tissue was allocated for histological study, while the remaining half was designated for biochemical investigation. The kidney tissues designated for histological analysis were preserved in a 10% formaldehyde solution for 24 h. After this period, the tissues subjected to standard histological techniques were embedded in paraffin.

### Hematoxylin–eosin and Picro Sirius staining

Hematoxylin–eosin (HxE) staining was performed on 5 µm thick sections derived from paraffin-embedded samples to elucidate the overall histological architecture and structural alterations of the kidney (YÖRÜKOĞLU K, 2021; Mostafa et al. 2021). Following deparaffinization and dehydration, the slices were stained with hematoxylin (MERCK®, 1.04302.0100) for 3 min and subsequently washed. Subsequently, they were immersed in a solution of acid alcohol followed by ammonia. The sections were subsequently stained with eosin (MERCK®, 1.15935.0025) for 1 min. Upon re-entering and completing the dehydration stage, the parts preserved in xylene for cleaning and clarification were subsequently coated with Entellan®. Picro Sirius Red (PSR) (GBL® CONVASTAIN, Picro Sirius Red Stain Kit, Ref No:5002, Türkiye) staining was conducted to assess the augmentation of connective tissue and hemorrhagic regions in the kidney. The sections were subjected to xylene and a sequence of decreasing alcohol concentrations, followed by immersion in Picro Sirius Red stain for 50 min. The sections, previously washed twice with buffer solution, underwent nuclear staining with Mayer’s hematoxylin for a duration of 5 min. Subsequent to being rinsed with running water, the portions were subjected to a succession of escalating alcohol concentrations and xylene, ultimately being encapsulated with Entellan®.

### Histometric measurements

Histometric measurements were conducted utilizing the LAS V4.8 image analysis software on a Leica® DM2500 LED research microscope equipped with an MC170 HD camera adapter. Measurements of the diameters of 20 glomeruli and renal corpuscles were acquired from serial sections of each animal in each group, utilizing an × 20 objective, based on preparations stained with HxE and PSR (Kotyk et al. 2016). The photographs of the requisite regions and the analyses derived from them were documented collectively.

### Immunohistochemical staining

Immunohistochemical staining of 5 µm thick slices mounted on poly L-lysine slides with a streptavidin–biotin-peroxidase complex (sABC) protocol (Duan et al. 2003) caspase-3 and −4 assays were conducted utilizing hydroxynonenal (4-HN) antibodies. Following deparaffinization, the sections were immersed in EDTA (DAKO, EnVisionFLEX DM848) for 30 min in a 600 W microwave oven for antigen retrieval. The sections were maintained in a 3% hydrogen peroxide solution formulated in methanol to inhibit endogenous peroxide activity and thereafter incubated in a protein block solution (Novocastra® Protein Block RE7102) for 15 min to avert non-specific binding. Caspase-3 (Cell Signaling Technology Inc.®, CST 9664S) and 4-HN antibodies (Biossusa®, BS6313R-TR) were utilized at a dilution of 1/400 (ScyTec Lab. Normal antibody diluent, ABB125) for 1 h at ambient temperature. After the antibody phase, sections were incubated in streptavidin-HRP conjugate (Novocastra® GERPN1231) solution for 5 min, followed by chromogen application using DAB (3–3′-diaminobenzidine, TA-060-HD, Thermo Fischer®) and counterstaining with Mayer’s hematoxylin. Sections were treated with a non-aqueous sealer (Entellan, Merck Millipore®) and analyzed using a Leica® DM2500 LED research microscope, with the results documented by photography and archived digitally.

### Detection of GSH-Px, SOD, MDA, TNF-α, and IL-6 in kidney tissues

Kidney tissues were homogenized in a phosphate buffer (25 mM, pH 7.4) utilizing IKA (Ultra Turrax T25 basic, IKA Labotechnik, Staufen, Germany) tissue grinders at 12,000 rpm and + 4 °C for 2–3 min on ice (Kahraman et al. 2020). The MDA concentration in homogenized tissues was assessed following the methodology established by Ohkawa (Ohkawa 1978). The reaction of thiobarbituric acid with MDA at 95 °C for 45 min yielded a pink chromogen in the media. The colored product was spectrophotometrically measured using a T80 UV/VIS spectrometer (PG Instruments Ltd.) at a wavelength of 535 nm. Results are presented as nanomoles per gram of moist tissue.

The supernatant was obtained by centrifuging homogenized tissue samples at 5000 rpm. Reduced glutathione peroxidase (GSH-Px) and superoxide dismutase (SOD) were analyzed in the supernatant. The GSH-Px level was evaluated following the methodology established by Ellman (Ellman 1959). The green-yellow hue resulted from the interaction of glutathione with 5,5′-dithiobis(2-nitrobenzoic acid) (DTNB). The colored product was spectrophotometrically quantified at 412 nm, and the results were reported as nmol/g of moist tissue.

The activity of the SOD enzyme was assessed following the methodology established by Sun et al. (Sun et al. 1988). Superoxide radicals that diminish nitro blue tetrazolium (NBT) were generated through the xanthine-xanthine oxidase interaction, resulting in the formation of a blue formazan compound. The colored product was spectrophotometrically quantified at 560 nm, and the data were represented as U/g protein.

Proinflammatory cytokine levels, including tumor necrosis factor-alpha (TNF-α) and interleukin-6 (IL-6), were assessed utilizing ELISA reader kits from kidney homogenate.

### Determination of BUN and creatinine in serum

To assess renal hemodynamics, blood samples were obtained from the left ventricle of the heart, collected in heparinized tubes, and subsequently centrifuged for 15 min at 4000 rpm at + 4 °C to isolate plasma (Altinoz et al. 2024). Plasma blood urea nitrogen (BUN) and creatinine levels were quantified utilizing Architect c 1600 automatic analyzer kits (Abbott, Abbott Park, IL, USA).

### Statistical analysis

Statistical data analysis was conducted using IBM SPSS Statistics version 25.0 for Windows software. The Shapiro–Wilk test indicated a normal distribution of the data (*P* > 0.05). Multiple comparisons were evaluated using a one-way ANOVA test with Tukey HSD correction. Results are expressed as mean ± standard deviation (SD), with *P* < 0.05 being statistically significant.

## Results

### Results regarding subjects’ live weights, renal corpuscle, and glomerulus diameters

**Fig. 1:**
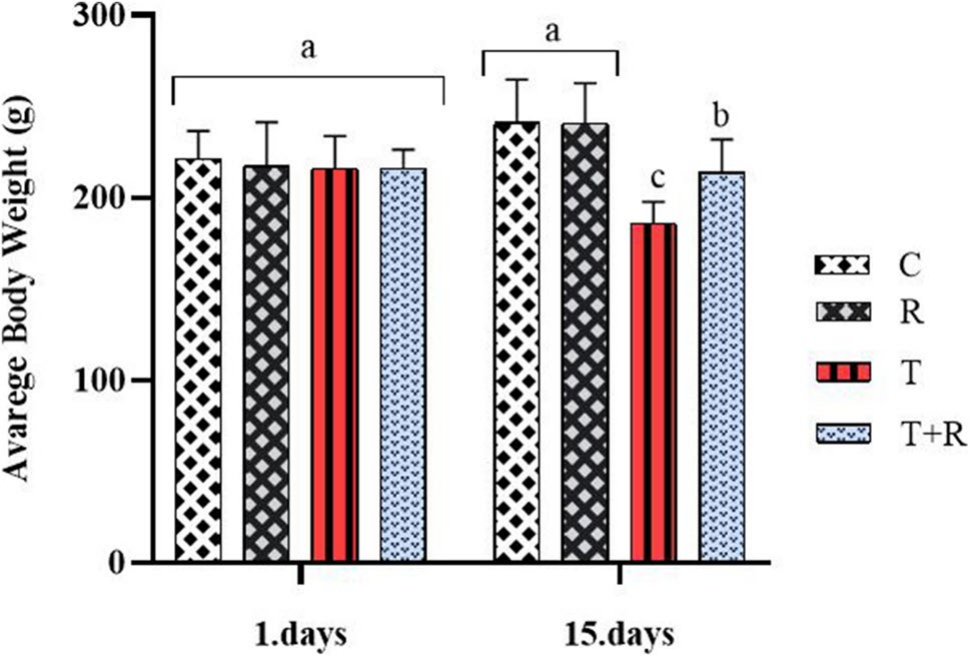
The statistical evaluation of the live body weights on the 1st and the 15th day of the experiment. 1. days: no statistically significant difference in the experimental groups’ average live body weight *P* > 0.05; 15. days: the average live body weight of group T was lower than other groups and statistically significant *P* < 0.05. The average live body weight of the T + R group was higher and statistically significant than that of the T group *P* < 0.05. There was no statistical difference between the C and R groups *P* > 0.05. ^a,b,c^: There is a statistically significant difference between groups with different letters on the figure (*P* < 0.05)

The assessment of the subjects’ live weights on the initial day of the trial revealed no statistically significant difference between the groups regarding live body weight (*P* > 0.05). The live body weight assessment conducted at the conclusion of the trial revealed a statistically significant reduction in the T group relative to the other groups (*P* < 0.05). At the conclusion of the trial, the C and R groups exhibited similar results; however, the T + R group demonstrated statistically significant differences from the other groups (*P* < 0.05, Fig. 1).

**Fig. 2:**
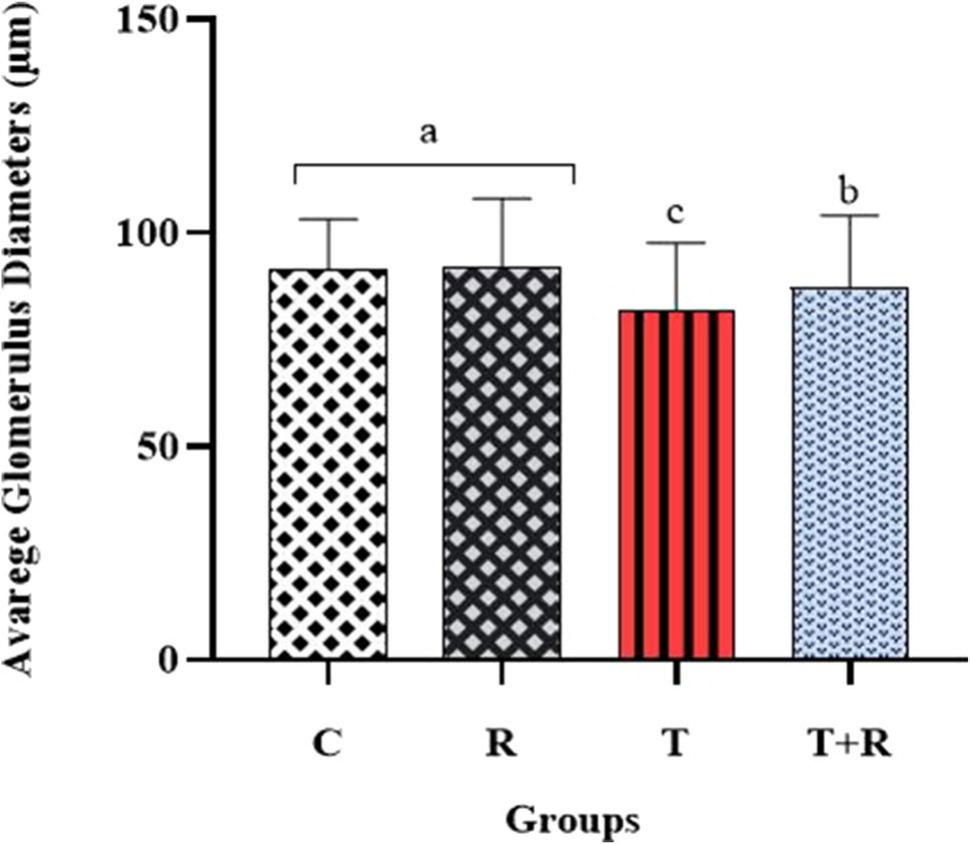
The statistical evaluation of the average glomerulus diameters of the groups. The average glomerulus diameter of group T was lower than other groups and statistically significant *P* < 0.05. The average glomerulus diameter of the T + R group was higher and statistically significant than that of the T group *P* < 0.05. There was no statistical difference between the C and R groups *P* > 0.05. ^a,b,c^: There is a statistically significant difference between groups with different letters on the figure (*P* < 0.05)

**Fig. 3:**
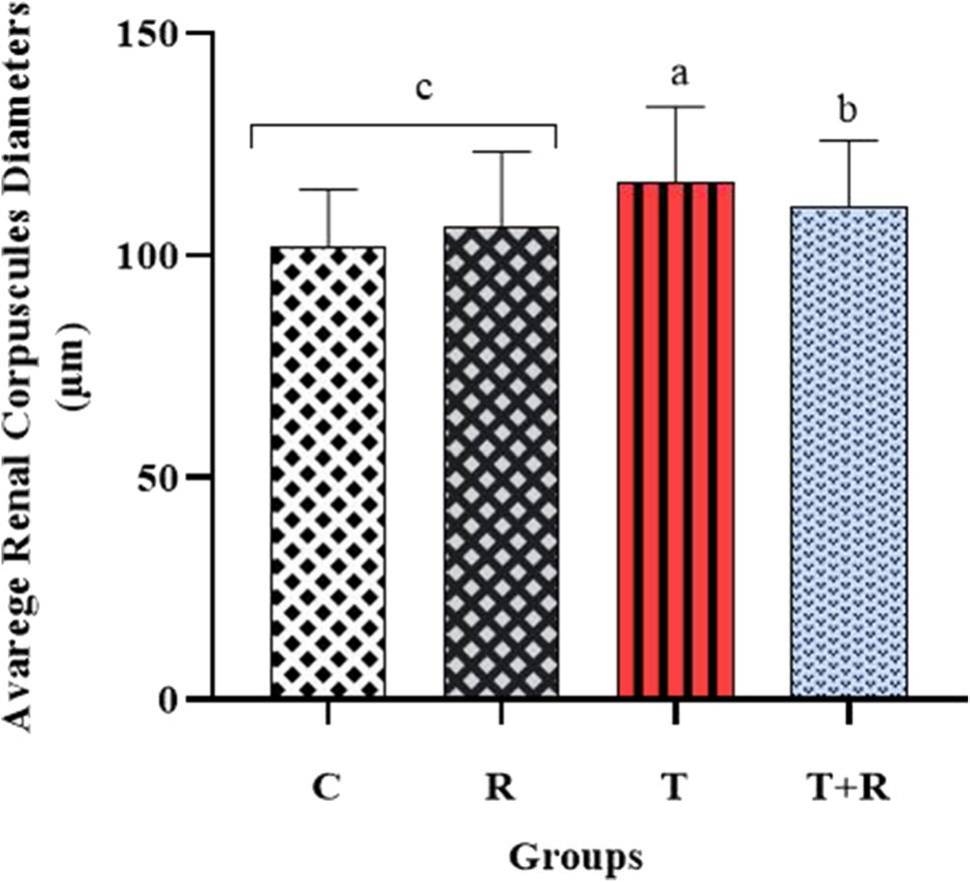
The statistical evaluation of the average renal corpuscle diameters of the groups. The average renal corpuscle diameter of group T was higher than other groups and statistically significant *P* < 0.05. The average renal corpuscle diameter of the T + R group was lower and statistically significant than that of the T group *P* < 0.05. There was no statistical difference between the C and R groups *P* > 0.05

The assessment of glomerulus diameters revealed a substantial reduction in the T group relative to the other groups (*P* < 0.05). The glomerulus diameters were found to be greater in the T + R group than in the T group (*P* < 0.05). No significant change in glomerulus diameter was detected between the C and R groups (*P* > 0.05). The examination of renal corpuscle diameters revealed that the T group had the biggest diameter, with statistically significant differences observed when compared to the other groups (*P* < 0.05). The renal corpuscle diameters in the T + R group were found to be significantly smaller than those in the T group (*P* < 0.05). The renal corpuscle sizes in the C and R groups were modest, with no statistically significant difference found (*P* > 0.05, Figs. 2 and 3).

### Histopathological results

**Fig. 4:**
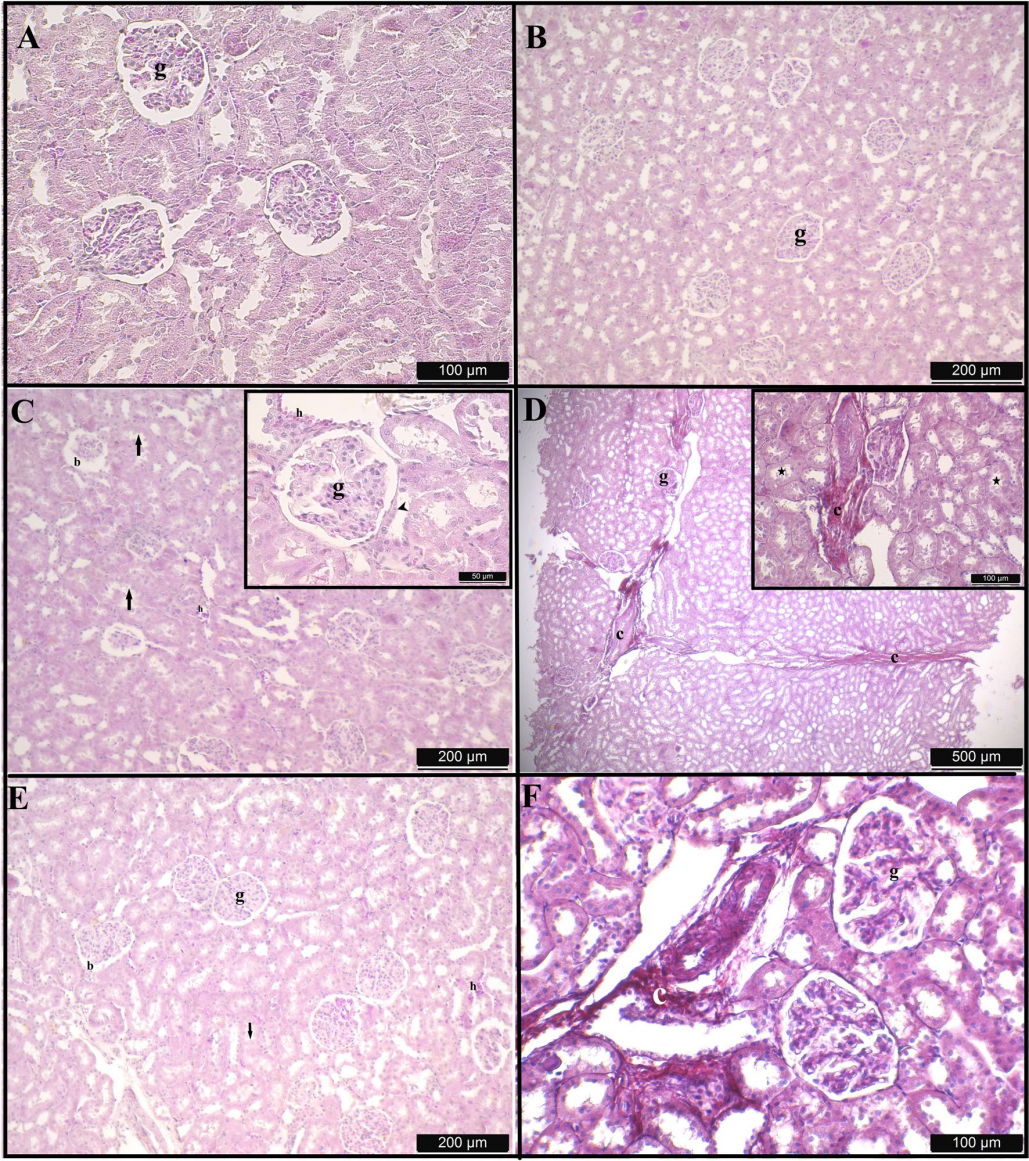
Sections obtained from kidney tissues of rats in the control (**A**), resveratrol (**B**), titanium (**C** and **D**), and titanium + resveratrol (**E** and **F**) groups. HxE and Picro Sirius stain. g: glomerulus; b: Bowman space; h: hemorrhage; arrow: exfoliated tubule cells; arrowhead: degenerated macula densa; c: increased connective tissue; star: edema in tubules

The examination of hematoxylin–eosin and Picro-Sirius red-stained preparations under light microscopy revealed histologically normal kidney structures in both the C and R groups. In the areas where the cortex-medulla differentiation was readily discernible in the preparations, the glomerular and tubular structures also had a normal histological appearance (Fig. 4A and B). The Bowman capsule and Bowman space were within normal parameters (Fig. 4A and B). The preparations derived from the tissues of the T group exhibited distorted and shrunken glomeruli, an enlarged Bowman space, and the presence of exfoliated cells within the tubules (Fig. 4C). Damage to the macula densa regions, lymphocytic infiltration, and hemorrhagic areas were observed throughout the tissue (Fig. 4C). Concurrently, there were regions where the connective tissue proliferated within the tissue (Fig. 4D). Upon evaluating the sections from the T + R group, it was seen that the glomerular structure was significantly better preserved compared to the T group (Fig. 4E). The width of the Bowman space resembled that of the C group; however, there were fewer hemorrhagic patches observed compared to the T group, although tubular exfoliation was comparable to the T group (Fig. 4E). The quantity of vacuolated cells was elevated in the tubules (Fig. 4E). Lymphocytic infiltration was absent (Fig. 4E). While regions of increased connective tissue were noted, a decrease was detected in comparison to the T group (Fig. 4F).

### Results of immunohistochemistry staining with caspase-3

**Fig. 5:**
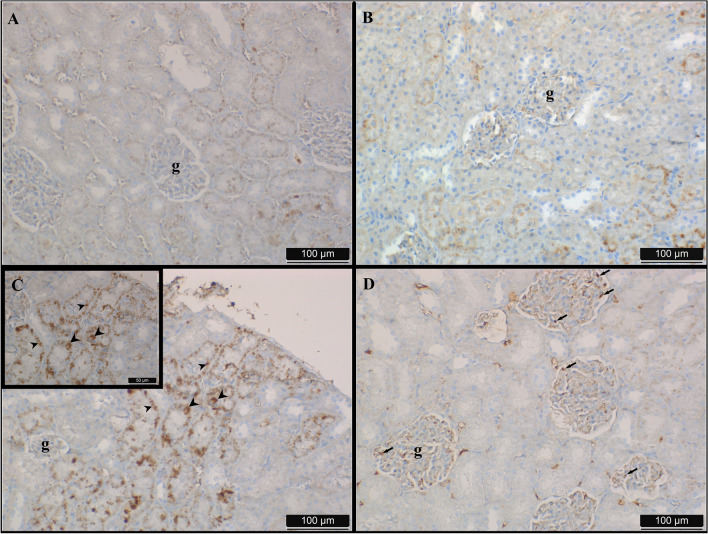
Caspase-3 immunostaining applied to sections obtained from kidney tissues of rats in the control (**A**), resveratrol (**B**), titanium (**C**), and titanium + resveratrol (**D**) groups. g: glomerulus; arrowhead: tubule cells showing positive immune reaction; arrow: podocytes showing positive immune reaction

Following staining with the caspase-3 antibody, comparable low-intensity immune responses were noted in groups C and R (Fig. 5A and B). The reaction was more pronounced in distal tubules compared to proximal tubules (Fig. 5A and B). In samples from group T, a minimal immune response was noted in the glomerulus; however, in the distal tubules, a pronounced immunological reaction with significant staining was observed in several cells (Fig. 5C). The apical engagement was noted in certain cells and nuclear involvement in others, although basolateral involvement was predominantly found (Fig. 5C). In group T + R, immunological reactions were predominantly localized in the glomerulus and podocytes, while being intracytoplasmic and markedly diminished in the tubules (Fig. 5D).

### Results of immunohistochemistry staining with 4-hydroxynonenal

**Fig. 6:**
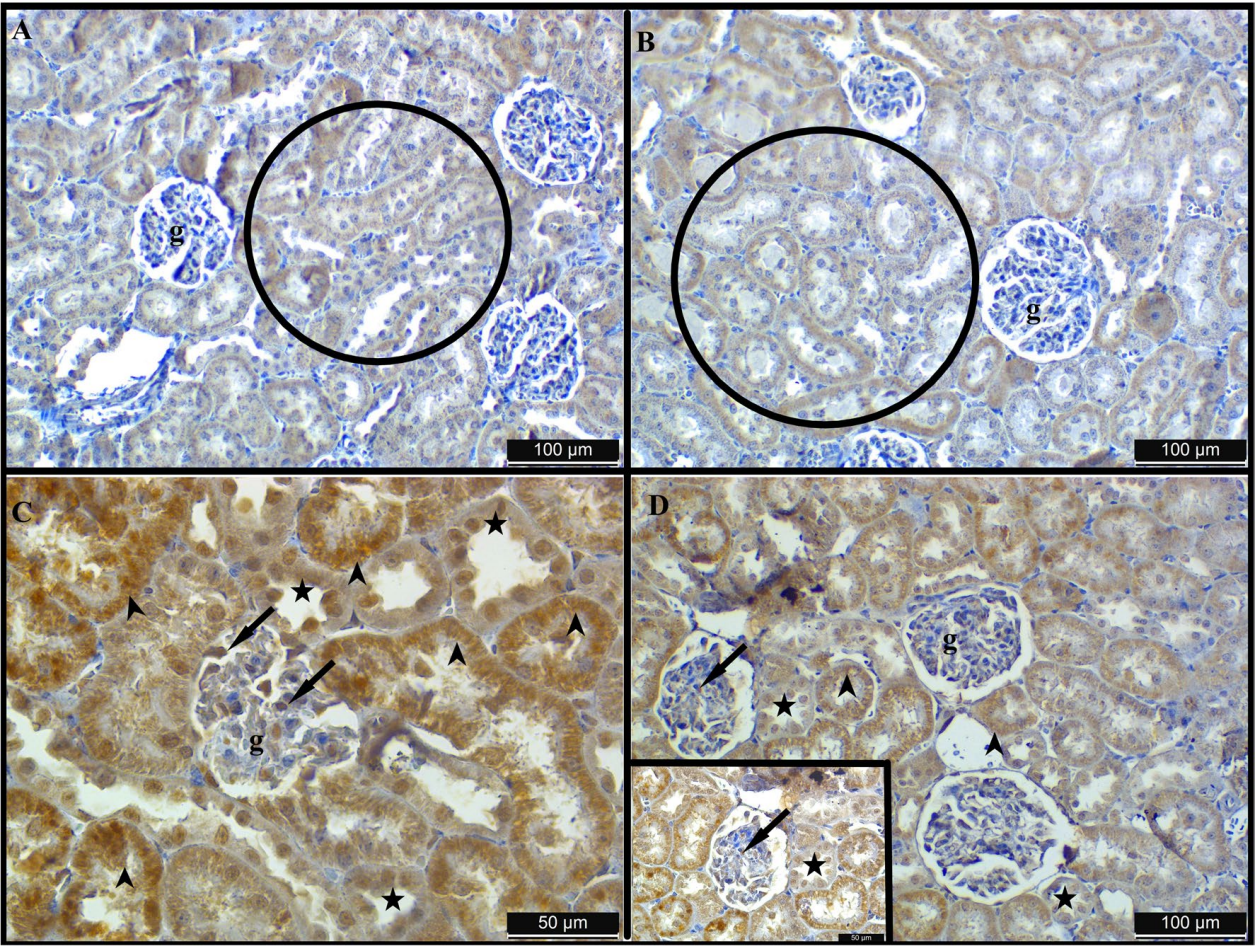
4-HN immunostaining applied to sections obtained from kidney tissues of rats in control (**A**), resveratrol (**B**), titanium (**C**), and titanium + resveratrol (**D**) groups. g: glomerulus; arrowhead: tubule cells showing intracytoplasmic positive immune reaction; arrow: podocytes showing positive immune reaction; black circle: tubule cells showing weak immune reaction; asterisk: intranuclear positive immune reaction in proximal tubules.

In preparations derived from groups C and R, the positive immunological response for 4-HN was shown to be minimal. No immune response was detected in the glomerulus and Bowman’s capsule (Fig. 6A and B). The diminished immune response identified in group R was noted in the membrane localization within the tubules, whereas intracytoplasmic and nuclear immune responses were absent (Fig. 6B). Intracytoplasmic immune reactions were noted in distal and extensive collective tubules, while nuclear immune reactions were identified in proximal tubules, as evidenced by sections from group T (Fig. 6C). A robust positive immunological response was detected in podocytes within the glomerulus and in the nuclei of Bowman’s capsule (Fig. 6C). The application of 4-HN immunostaining to sections from the T + R group revealed that the positive nuclear immune response in the proximal tubules was less pronounced than in the T group, the immune response in certain collective tubules was markedly diminished, and no response was detected in the distal tubules (Fig. 6D). Fewer podocytes in the glomerulus exhibited positive immunological reactions compared to the T group (Fig. 6D).

### Results regarding oxidative stress and inflammation markers in kidney tissues

**Fig. 7:**
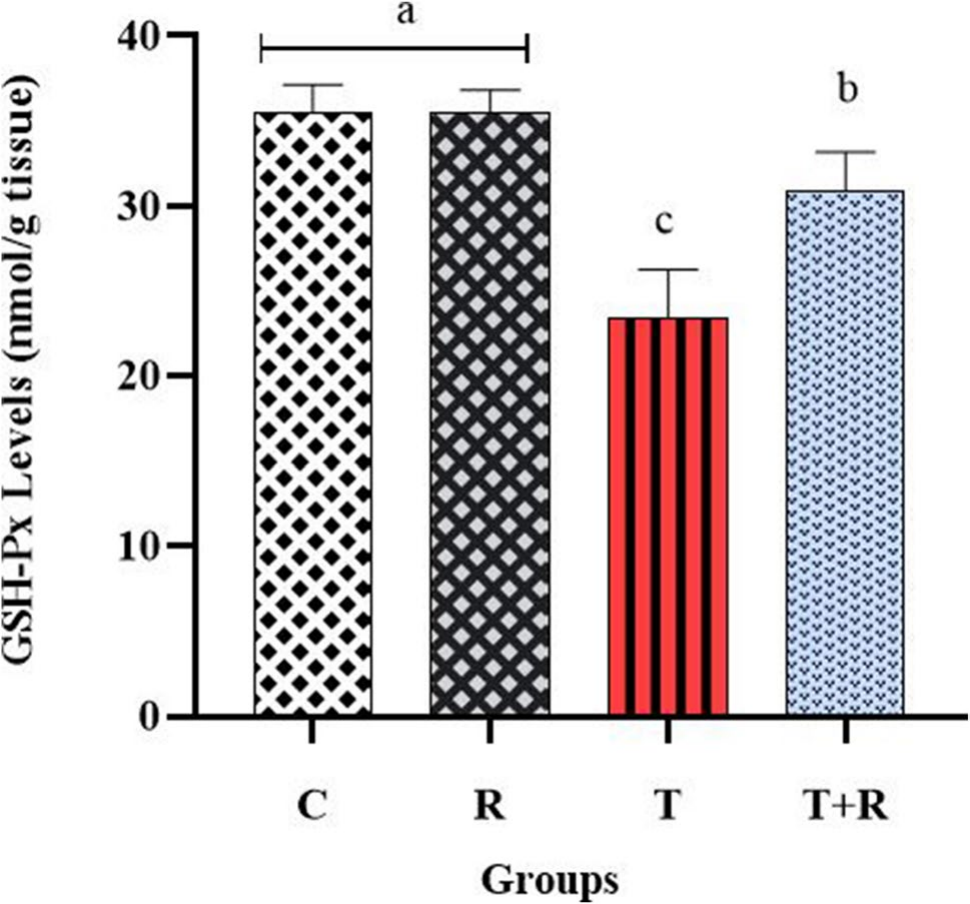
The statistical evaluation of the GSH-Px levels. The GSH-Px level of group T was lower than other groups and statistically significant *P* < 0.05. The GSH-Px level of the T + R group was higher and statistically significant than that of the T group *P* < 0.05. There was no statistical difference between the C and R groups *P* > 0.05

**Fig. 8:**
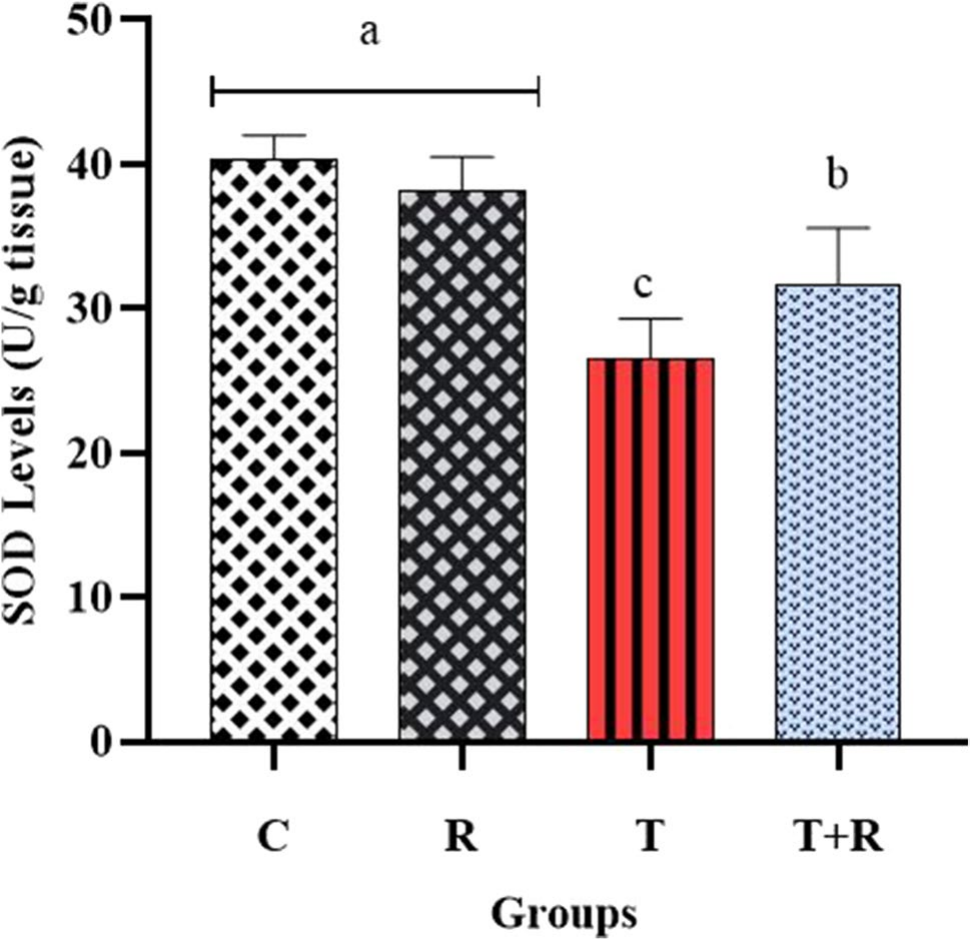
The statistical evaluation of the SOD levels The SOD level of group T was lower than other groups and statistically significant *P* < 0.05. The SOD level of the T + R group was higher and statistically significant than that of the T group *P* < 0.05. There was no statistical difference between the C and R groups *P* > 0.05

**Fig. 9:**
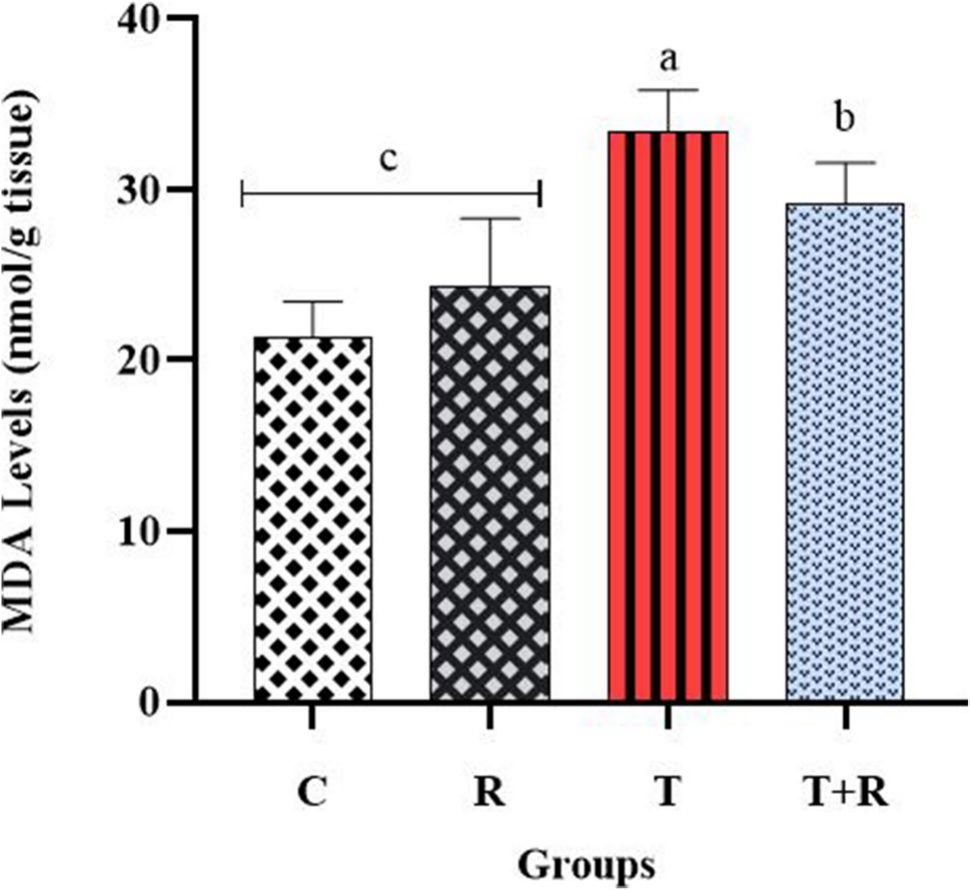
The statistical evaluation of the MDA levels. The MDA level of group T was higher than other groups and statistically significant *P* < 0.05. The MDA level of the T + R group was lower and statistically significant than that of the T group *P* < 0.05. There was no statistical difference between the C and R groups *P* > 0.05

The concentrations of GSH-Px, SOD, and MDA, recognized as biochemical indicators of oxidative stress in renal tissue, were assessed. The evaluation revealed that GSH-Px and SOD activities were significantly reduced in the T group compared to the other experimental groups, whereas MDA levels were significantly elevated in the T group relative to the other groups (*P* < 0.05). The activities of GSH-Px and SOD were seen to be elevated in the T + R group relative to the T group (*P* < 0.05). The levels of oxidative stress indicators were found to be comparable between the C and R groups, with no statistically significant differences (*P* > 0.05) (Figs. 7, 8, 9).

**Fig. 10:**
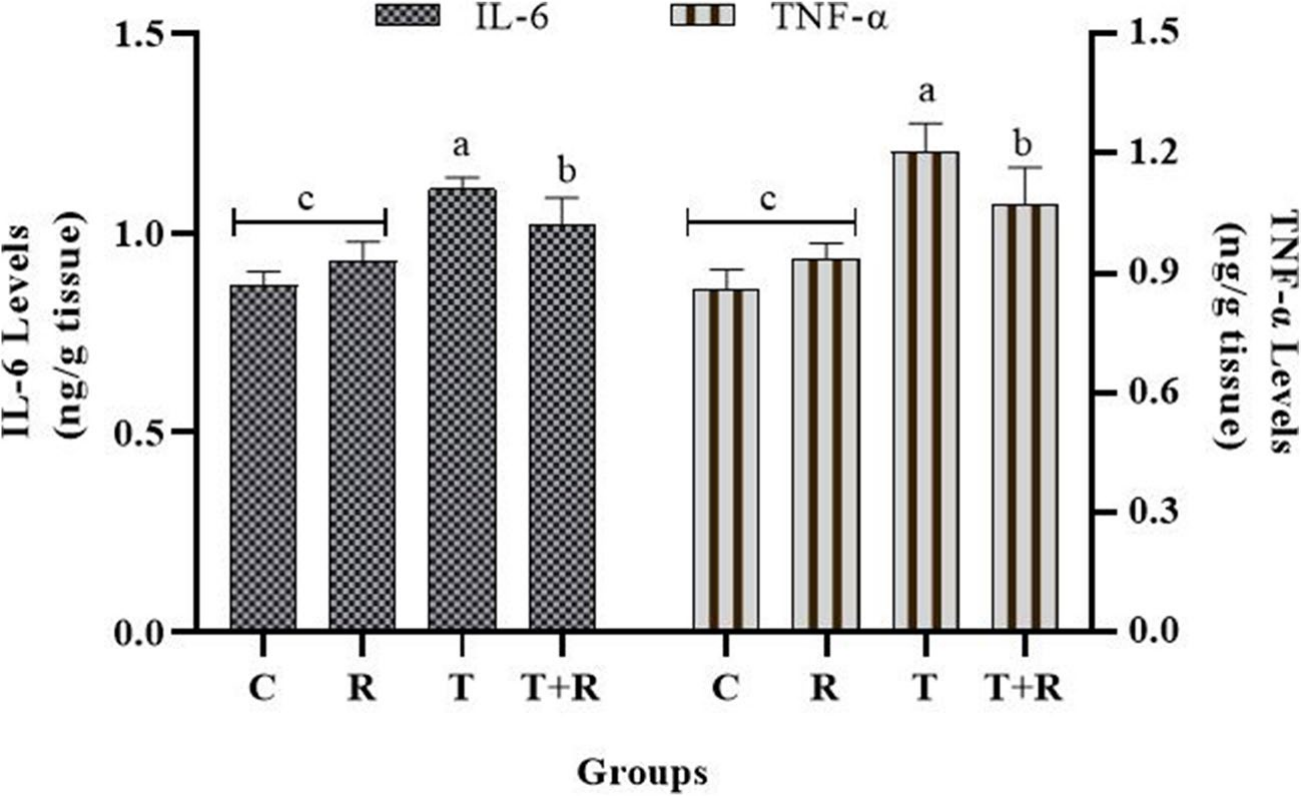
The statistical evaluation of the IL-6 and TNF-α levels. The TNF-α and IL-6 levels of group T were higher than other groups and statistically significant *P* < 0.05. The TNF-α and IL-6 levels of the T + R group were lower and statistically significant than that of the T group *P* < 0.05. There was no statistical difference between the C and R groups *P* > 0.05

The analysis of TNF-α and IL-6 levels, recognized as inflammation markers, revealed a substantial increase in IL-6 and TNF-α levels in the T group relative to the other groups (*P* < 0.05), and a statistically significant decrease in the T + R group compared to the T group (*P* < 0.05). No statistical difference was found between groups C and R (*P* > 0.05) (Fig. 10).

### Serum BUN and creatinine results

**Fig. 11:**
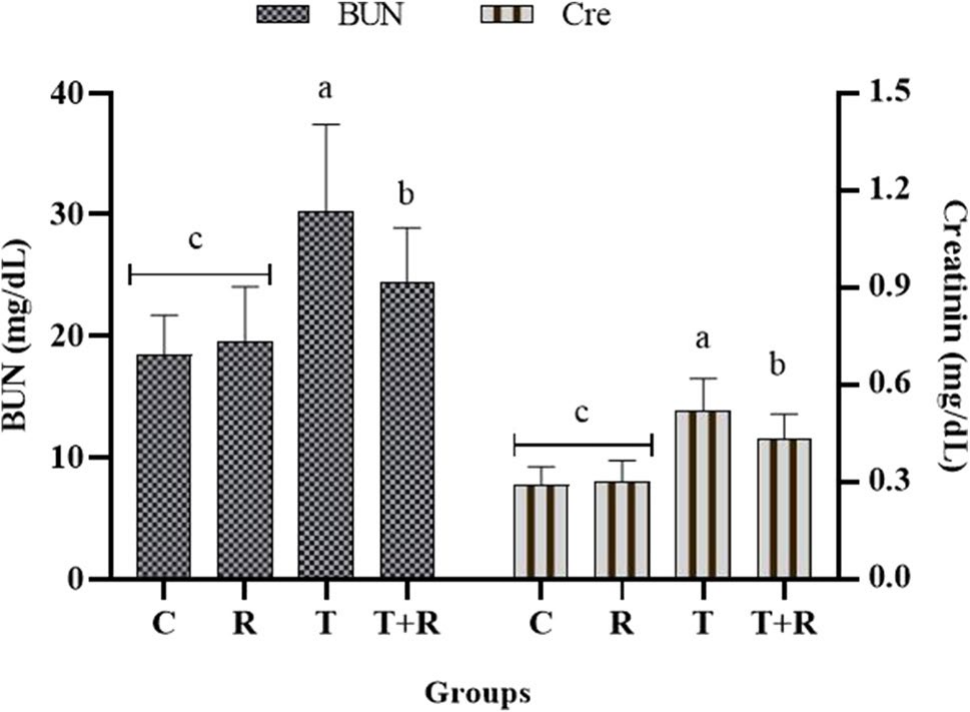
The statistical evaluation of the serum BUN and creatinine levels. The BUN and creatinine levels of group T were higher than other groups and statistically significant *P* < 0.05. The BUN and creatinine levels of the T + R group were lower and statistically significant than that of the T group *P* < 0.05. There was no statistical difference between the C and R groups *P* > 0.05

The assessment of BUN and creatinine levels in the subjects’ sera revealed that both levels were statistically significantly elevated in the T group relative to the other three groups (*P* < 0.05), whereas these levels were reduced in the T + R group compared to the T group. No statistically significant difference was seen between the C and R groups, with levels being comparable (*P* > 0.05) (Fig. 11).

## Discussion

Titanium dioxide (E171), utilized globally primarily as a food additive, is regarded as an inert and harmless substance (Wang et al. 2013). Titanium dioxide’s propensity to accumulate in internal organs and contribute to mechanisms resulting in organ damage has positioned this substance prominently in cytotoxicity and genotoxicity research.

It is established that a balance exists between oxidants and antioxidants in physiological settings (Erdemli et al. 2017). Reports indicate that TiO_2_ induces oxidative stress by the generation of reactive oxygen species (ROS), and also activates several signaling pathways, leading to apoptotic and proinflammatory responses (Bautista-Pérez et al. 2024). The tissue damage resulting from oxidative stress can be biochemically assessed through the activities of antioxidant enzymes, specifically superoxide dismutase (SOD) and glutathione peroxidase (GSH-Px) (Erdemli et al. 2017), as well as by measuring lipid peroxidation products, including malondialdehyde (MDA) levels (Guentsch et al. 2008). Titanium dioxide has been identified in several organs including kidneys following inhalation, intraperitoneal, or oral exposure (Bautista-Pérez et al. 2024; Parashar et al. 2024).

Titanium dioxide is absorbed from the gastrointestinal system and disseminated to other organs in this manner (Ferrante et al. 2023). Numerous investigations involving rats and mice have demonstrated that TiO_2_ can traverse the intestinal barrier, subsequently causing hypertrophy and hyperplasia in the affected tissues (Xiao et al. 2024; Bettini et al. 2017). A study assessing the protective effects of quercetin and idebenone against hepatotoxicity induced by TiO_2_ in rats revealed a substantial rise in MDA levels in the TiO_2_ group relative to the control group. This investigation indicated that substantial elevations in TNF-α and IL-6 levels were noted in the TiO_2_ poisoning group, potentially leading to metabolic illnesses and immunological disorders (Fadda et al. 2018). A study administering TiO_2_ intratracheally to mice found lung damage defined by oxidative stress and alterations in proinflammatory cytokines, and this work also shows that exposure to TiO_2_ can greatly enhance the expression of TNF-α and IL-6 (Sun et al. 2012). The concentrations of proinflammatory cytokines, including TNF-α and IL-6, which are recognized to elevate during inflammation, were shown to be statistically substantially greater in the T group relative to the other groups in this investigation (*P* < 0.05).

Studies of TiO_2_-induced nephrotoxicity revealed that TiO_2_ elevated MDA levels and kidney function markers BUN and creatinine levels while diminishing serum SOD and GPX levels (Shirdare et al. 2022; Morgan et al. 2017). In our investigation, the nephrotoxicity model established in rats supplied TiO_2_ via oral intragastric gavage revealed a significant elevation in MDA levels, alongside a reduction in SOD and GPX levels in the TiO_2_-treated rats (*P* < 0.05), the assessment of BUN and creatinine levels in blood serum revealed statistically significant increases in the T group compared to the other groups (*P* < 0.05), consistent with the aforementioned studies.

A study examining TiO_2_-induced renal damage, administered at a dosage of 50 mg/kg via intragastric gavage for 7 days in rats, revealed a significant increase in MDA concentration in the kidney tissue of the TiO_2_ group, accompanied by a notable decrease in SOD activity. These alterations were linked to oxidative stress in renal tissue. In the present investigation, it was established that the concentrations of creatinine and BUN in the blood greatly increased, while the weights of the kidneys and body were reduced in the animals fed TiO_2_. The alteration in kidney weight may stem from damage to the proximal tubule and glomerulus, while the reduction in body weight could be attributed to the nephrotoxic effects of TiO_2_ and its capacity to induce apoptosis in other tissues (Alidadi et al. 2018). Our work posits that the reductions in live weights noted in the TiO_2_ group may be attributable to the nephrotoxic effects of TiO_2_, as supported by existing literature.

The kidney is regarded as one of the most susceptible organs to toxic agents in the body owing to its elevated blood flow and capacity to concentrate waste products (L'azou et al. 2002). The harmful effects of TiO_2_ are linked to histopathological and functional alterations in various organs, including the kidneys, and may induce apoptotic responses (Bautista-Pérez et al. 2024). The administration of TiO_2_ led to various myocardial cytomorphic alterations, including structurally disorganized, degenerated, and apoptotic cardiomyocytes, and these alterations stemmed from the oxidative burden on cardiomyocytes (El-Din et al. 2019). Numerous investigations indicate that TiO_2_ accumulates predominantly in kidneys, resulting in histological damage to these tissues and alterations in biochemical markers (Morgan et al. 2017; Bakour et al. 2021). A study characterized the histological alterations induced by TiO_2_ in kidney tissue as degeneration of proximal tubules, induction of apoptosis in these tubules, reduction in glomerular diameters, and infiltration of inflammatory cells (Alidadi et al. 2018). In another study oxidative stress induced by TiO_2_ revealed vascular congestion and perivascular mononuclear cell infiltration in kidney tissue (Hassanein and El-Amir 2017). This study observed that TiO_2_ compromised the structural integrity of the glomerulus and tubules in the kidney, resulting in notable histological abnormalities. The glomerulus diameter decreased in the T group, but it increased in the T + R group, nearing the dimensions of the control group (*P* < 0.05). The renal corpuscle diameter was significantly enlarged in the T group and showed a tendency to diminish in the T + R group, while the smallest diameters were seen in the control and resveratrol groups (*P* < 0.05). In this study, the preparations obtained from the T group tissues had deformed and undersized glomeruli, an expanded Bowman space, and exfoliated cells inside the tubules. Damage to the macula densa regions, lymphocytic infiltration, and hemorrhagic areas were noted throughout the tissue. Simultaneously, there were areas where the connective tissue expanded within the tissue. The current observations align with earlier histological investigations indicating that the nephrotoxic effect is associated with TiO_2_ exposure (Alidadi et al. 2018; Abdel-Halim et al. 2022).

Reports indicate that the activation of oxidative stress in the kidneys may disturb the balance between inflammatory stimuli and the cellular antioxidant defense mechanism, potentially leading to pathophysiological disorders and apoptosis (Molaei et al. 2021). Apoptosis is a multifaceted biological process essential for regulating cell survival by removing damaged or diseased cells (Kuranaga 2012). 4-HN has been documented to promote apoptotic cell death by modulating the expression of proteins associated with apoptosis activated by stressors, including cell death signaling pathways and oxidative agents (Ji et al. 2016). The apoptotic response elicited by 4-HN has been documented to activate caspases (West et al. 2004), with caspase-3 protein expression identified as a significant apoptotic marker (Abdel-Halim et al. 2022). Titanium dioxide has been demonstrated to induce apoptosis through the activation of caspase-3 in animal models (Abbasi-Oshaghi et al. 2019). One report revealed that the administration of TiO_2_ to mice over 14 days resulted in pathological damage, characterized by heightened oxidative stress, DNA damage, elevated pro-apoptotic factors (BAX, caspase-3/9, p53), and diminished anti-apoptotic factor (Bcl-2) (Shukla et al. 2014). The data indicate that TiO_2_ can trigger cell death via the caspase-dependent signaling pathway.

This investigation revealed that, as a result of caspase-3 staining, the T group exhibited a significantly elevated number of apoptotic cells. In samples from group T, a negligible immune response was found in the glomerulus; nevertheless, in the distal tubules, a marked immunological reaction with substantial staining was noted in several cells. Apical engagement was observed in specific cells, while nuclear participation was noted in others; nonetheless, basolateral involvement was primarily identified. The cells responding with 4-HN in the T group were abundant and had intense staining throughout the tissue. Intracytoplasmic immune reactions were observed in distal and extensive collecting tubules, whereas nuclear immune reactions were detected in proximal tubules of group T. A strong favorable immunological response was observed in podocytes within the glomerulus and in the nuclei of Bowman’s capsule. Nonetheless, this circumstance led to diminished staining intensity in the T + R group for both 4-HN and caspase-3 immunoreactions.

Numerous investigations have shown that the importance of antioxidants is unequivocal, particularly in mitigating oxidative stress in the prevention and treatment of TiO_2_-induced organ damage (Mihailovic et al. 2021). In this context, phytochemicals like resveratrol have gained prominence in research for their capacity to mitigate TiO_2_ toxicity owing to their antioxidant, anti-inflammatory, and anti-apoptotic characteristics (Ryu et al. 2016). Resveratrol has been demonstrated to offer protection against oxidative stress, a primary contributor to numerous illnesses, via multiple redox-related molecular mechanisms; moreover, both in vivo and in vitro research have illustrated the anti-inflammatory characteristics of resveratrol, which are ascribed to its capacity to diminish the generation of inflammatory mediators (Kheira et al. 2024). A study indicated that resveratrol lowers IL-6 and TNF-α levels, a process associated with its anti-inflammatory effects (Fuggetta et al. 2016). Numerous research assessing the protective effects of resveratrol against toxicity have yielded remarkable results, demonstrating its capacity to inhibit apoptosis (Yalcın et al. 2024; Son et al. 2014). This study observed that resveratrol had protective and restorative effects against TiO_2_-induced nephrotoxicity. We determined that these protective benefits may result from decreased oxidative stress and lipid peroxidation, suppression of inflammation, and enhanced anti-apoptotic activity.

This study, the inaugural investigation into the association between TiO_2_ (E171) and resveratrol in rats, demonstrated TiO_2_ nephrotoxicity through alterations in histopathological and biochemical markers in the kidney, resulting in oxidative stress. The concurrent administration of resveratrol and TiO_2_ markedly mitigated the majority of TiO_2_-induced alterations in the kidney, encompassing apoptotic and proinflammatory responses, antioxidant dysregulation, serum renal function metrics, and histopathological changes. The use of resveratrol effectively mitigated the harmful effects of TiO_2_.

Consequently, resveratrol is believed to exert an inhibitory and protective effect against TiO_2_ nephrotoxicity and may serve as an antidote to TiO_2_ toxicity.

## Limitations of the study and future research

This study has certain limitations that must be acknowledged. This study might have benefited by testing various doses and application durations of both TiO_2_ and resveratrol to determine if substantial changes in the outcomes would occur. Nevertheless, this proved unfeasible in the study owing to financial limitations. Secondly, given the primary exposure route in the trial was oral, intragastric gavage was employed, making it essential to assess the renal implications of various exposure routes. The resveratrol utilized in the study was procured in its pure form from the manufacturer of the chemical compound and subsequently analyzed. We assert that the findings derived from the utilization of natural or commonly accessible dietary supplements will enhance the existing literature.

## Data Availability

All source data for this work (or generated in this study) are available upon reasonable request.

## References

[CR1] Abbasi-Oshaghi E, Mirzaei F, Pourjafar M (2019) NLRP3 inflammasome, oxidative stress, and apoptosis induced in the intestine and liver of rats treated with titanium dioxide nanoparticles: in vivo and in vitro study. Int J Nanomedicine 14:1919–1936 30936694 10.2147/IJN.S192382 PMC6421874

[CR2] Abdel-Halim KY, Osman SR, Abuzeid MAF, El-Danasoury HTM, Khozimy AM (2022) Apoptotic and histopathological defects enhanced by titanium dioxide nanoparticles in male mice after short-term exposure. Toxicol Rep 9:1331–1346 36518392 10.1016/j.toxrep.2022.06.003 PMC9743451

[CR3] Alidadi H, Khorsandi L, Shirani M (2018) Effects of quercetin on tubular cell apoptosis and kidney damage in rats induced by titanium dioxide nanoparticles. Malays J Med Sci 25:72–81 30918457 10.21315/mjms2018.25.2.8 PMC6422581

[CR4] Altinoz E, Cetinavci D, Abdulkareem Aljumaily SA, Elbe H, Cengil O and Bicer Y (2024) Crocin, the compound of the dried stigma of Crocus sativus L (saffron), restores doxorubicin-induced disturbances in kidney functioning, oxidative stress, inflammation, renal tissue morphology and TGF-β signalling pathways. Nat Product Res 1–14 10.1080/14786419.2024.2344180 38662441

[CR5] Ayorinde T and Sayes SCM (2023) An updated review of industrially relevant titanium dioxide and its environmental health effects. J Hazard Mater Lett 100085

[CR6] Bakour M, Hammas N, Laaroussi H, Ousaaid D, Fatemi HE, Aboulghazi A, Soulo N, Lyoussi B (2021) Moroccan bee bread improves biochemical and histological changes of the brain, liver, and kidneys induced by titanium dioxide nanoparticles. Biomed Res Int 2021:6632128 34258274 10.1155/2021/6632128 PMC8249149

[CR7] Bautista-Pérez R, Cano-Martínez A, Herrera-Rodríguez MA, Ramos-Godinez MdP, Pérez Reyes OL, Chirino YI, Rodríguez Serrano ZJ, López-Marure R (2024) Oral exposure to titanium dioxide E171 and zinc oxide nanoparticles induces multi-organ damage in rats: role of ceramide. Int J Mol Sci 25:5881 38892068 10.3390/ijms25115881 PMC11172338

[CR8] Bettini S, Boutet-Robinet E, Cartier C, Coméra C, Gaultier E, Dupuy J, Naud N, Taché S, Grysan P, Reguer S (2017) Food-grade TiO2 impairs intestinal and systemic immune homeostasis, initiates preneoplastic lesions and promotes aberrant crypt development in the rat colon. Sci Rep 7:40373 28106049 10.1038/srep40373 PMC5247795

[CR9] Brand W, Peters RJ, Braakhuis HM, Maślankiewicz L, Oomen AG (2020) Possible effects of titanium dioxide particles on human liver, intestinal tissue, spleen and kidney after oral exposure. Nanotoxicology 14:985–1007 32619159 10.1080/17435390.2020.1778809

[CR10] Chandra K, Salman AS, Mohd A, Sweety R, Ali KN (2015) Protection against FCA induced oxidative stress induced DNA damage as a model of arthritis and in vitro anti-arthritic potential of Costus speciosus rhizome extract. Int J Pharm Phytopharmacol Res 7:383–389

[CR11] Chang X, Heene E, Qiao F, Nick P (2011) The phytoalexin resveratrol regulates the initiation of hypersensitive cell death in Vitis cell. PLoS ONE 6:e26405 22053190 10.1371/journal.pone.0026405 PMC3203900

[CR12] Coşkun G, Özgür H (2011) Apoptoz ve nekrozun moleküler mekanizması. Arşiv Kaynak Tarama Dergisi 20:145–158

[CR13] Duan WR, Garner DS, Williams SD, Funckes-Shippy CL, Spath IS, Blomme EA (2003) Comparison of immunohistochemistry for activated caspase-3 and cleaved cytokeratin 18 with the TUNEL method for quantification of apoptosis in histological sections of PC-3 subcutaneous xenografts. J Pathol: A J Pathol Soc Great Brit Irel 199:221–228 10.1002/path.1289 12533835

[CR14] El-Din EAA, Mostafa HE-S, Samak MA, Mohamed EM, El-Shafei DA (2019) Could curcumin ameliorate titanium dioxide nanoparticles effect on the heart? A histopathological, immunohistochemical, and genotoxic study. Environ Sci Pollut Res 26:21556–21564 10.1007/s11356-019-05433-2 31127514

[CR15] Elgrabli D, Beaudouin R, Jbilou N, Floriani M, Pery A, Rogerieux F, Lacroix G (2015) Biodistribution and clearance of TiO2 nanoparticles in rats after intravenous injection. PLoS ONE 10:e0124490 25909957 10.1371/journal.pone.0124490 PMC4409301

[CR16] Ellman GL (1959) Tissue sulfhydryl groups. Arch Biochem Biophys 82:70–77 13650640 10.1016/0003-9861(59)90090-6

[CR17] Erdemli ME, Gul M, Altinoz E, Zayman E, Aksungur Z, Bag HG (2017) The protective role of crocin in tartrazine induced nephrotoxicity in Wistar rats. Biomed Pharmacother 96:930–935 29217164 10.1016/j.biopha.2017.11.150

[CR18] Esterbauer H and Cheeseman KH (1990) [42] Determination of aldehydic lipid peroxidation products: malonaldehyde and 4-hydroxynonenal. Methods in enzymology, Elsevier, pp 407–421 10.1016/0076-6879(90)86134-h 2233308

[CR19] Fadda LM, Hagar H, Mohamed AM, Ali HM (2018) Quercetin and idebenone ameliorate oxidative stress, inflammation, DNA damage, and apoptosis induced by titanium dioxide nanoparticles in rat liver. Dose-Response 16:1559325818812188 30559635 10.1177/1559325818812188 PMC6291876

[CR20] Ferrante M, Grasso A, Salemi R, Libra M, Tomasello B, Fiore M, Copat C (2023) DNA damage and apoptosis as in-vitro effect biomarkers of titanium dioxide nanoparticles (TiO2-NPs) and the food additive E171 toxicity in colon cancer cells: HCT-116 and Caco-2. Int J Environ Res Public Health 20:2002 36767368 10.3390/ijerph20032002 PMC9915631

[CR21] Fuggetta MP, Bordignon V, Cottarelli A, Macchi B, Frezza C, Cordiali-Fei P, Ensoli F, Ciafrè S, Marino-Merlo F, Mastino A (2016) Downregulation of proinflammatory cytokines in HTLV-1-infected T cells by resveratrol. J Exp Clin Cancer Res 35:1–9 27448598 10.1186/s13046-016-0398-8 PMC4957876

[CR22] Geraets L, Oomen AG, Krystek P, Jacobsen NR, Wallin H, Laurentie M, Verharen HW, Brandon EF, de Jong WH (2014) Tissue distribution and elimination after oral and intravenous administration of different titanium dioxide nanoparticles in rats. Part Fibre Toxicol 11:1–21 24993397 10.1186/1743-8977-11-30 PMC4105399

[CR23] Guentsch A, Preshaw PM, Bremer-Streck S, Klinger G, Glockmann E, Sigusch BW (2008) Lipid peroxidation and antioxidant activity in saliva of periodontitis patients: effect of smoking and periodontal treatment. Clin Oral Invest 12:345–352 10.1007/s00784-008-0202-z 18509684

[CR24] Gui S, Li B, Zhao X, Sheng L, Hong J, Yu X, Sang X, Sun Q, Ze Y, Wang L (2013) Renal injury and Nrf2 modulation in mouse kidney following chronic exposure to TiO2 nanoparticles. J Agric Food Chem 61:8959–8968 23968166 10.1021/jf402387e

[CR25] Hassanein KMA, El-Amir YO (2017) Protective effects of thymoquinone and avenanthramides on titanium dioxide nanoparticles induced toxicity in Sprague-Dawley rats. Pathol - Res Pract 213:13–22 27916297 10.1016/j.prp.2016.08.002

[CR26] Ji Y, Dai Z, Wu G, Wu Z (2016) 4-Hydroxy-2-nonenal induces apoptosis by activating ERK1/2 signaling and depleting intracellular glutathione in intestinal epithelial cells. Sci Rep 6:32929 27620528 10.1038/srep32929 PMC5020658

[CR27] Kahraman T, Berköz M, Allahverdiyev O, Mahmood EA, Yıldırım M, Yalın S (2020) Can Juniperus communis L. oil improve nephropathy in diabetic rats. Clin Exp Health Sci 10:120–124

[CR28] Kawada N, Seki S, Inoue M, Kuroki T (1998) Effect of antioxidants, resveratrol, quercetin, and N-acetylcysteine, on the functions of cultured rat hepatic stellate cells and Kupffer cells. Hepatology 27:1265–1274 9581680 10.1002/hep.510270512

[CR29] Kheira HS, Elsayed GR, El-Adl M (2024) Liraglutide and resveratrol alleviated cyclosporin A induced nephrotoxicity in rats through improving antioxidant status, apoptosis and pro-inflammatory markers. Biochem Biophys Res Commun 730:150337 38986220 10.1016/j.bbrc.2024.150337

[CR30] Kirkland D, Aardema MJ, Battersby RV, Beevers C, Burnett K, Burzlaff A, Czich A, Donner EM, Fowler P, Johnston HJ (2022) A weight of evidence review of the genotoxicity of titanium dioxide (TiO2). Regul Toxicol Pharmacol 136:105263 36228836 10.1016/j.yrtph.2022.105263

[CR31] Kitada M, Koya D (2013) Renal protective effects of resveratrol. Oxid Med Cell Longev 2013:568093 24379901 10.1155/2013/568093 PMC3863562

[CR32] Kitada M, Kume S, Imaizumi N, Koya D (2011) Resveratrol improves oxidative stress and protects against diabetic nephropathy through normalization of Mn-SOD dysfunction in AMPK/SIRT1-independent pathway. Diabetes 60:634–643 21270273 10.2337/db10-0386 PMC3028365

[CR33] Kong L, Sun N, Wei L, Zhang L, Chen Y, Chang L, Su X (2020) Melatonin protects against myocardial ischemia-reperfusion injury by inhibiting contracture in isolated rat hearts. Nan Fang Yi Ke Da Xue Xue Bao 40:958–964 32895155 10.12122/j.issn.1673-4254.2020.07.07 PMC7386215

[CR34] Kotyk T, Dey N, Ashour AS, Balas-Timar D, Chakraborty S, Ashour AS, Tavares JMRS (2016) Measurement of glomerulus diameter and Bowman’s space width of renal albino rats. Comput Methods Programs Biomed 126:143–153 26796351 10.1016/j.cmpb.2015.10.023

[CR35] Kuranaga E (2012) Beyond apoptosis: caspase regulatory mechanisms and functions in vivo. Genes Cells 17:83–97 22244258 10.1111/j.1365-2443.2011.01579.x

[CR36] L’azou B, Henge-Napoli M-H, Minaro L, Mirto H, Barrouillet M-P, Cambar J (2002) Effects of cadmium and uranium on some in vitro renal targets. Cell Biol Toxicol 18:329–340 12240964 10.1023/a:1019536115152

[CR37] Meena R, Rani M, Pal R, Rajamani P (2012) Nano-TiO2-induced apoptosis by oxidative stress-mediated DNA damage and activation of p53 in human embryonic kidney cells. Appl Biochem Biotechnol 167:791–808 22614867 10.1007/s12010-012-9699-3

[CR38] Mihailovic V, Katanic Stankovic JS, Selakovic D, Rosic G (2021) An overview of the beneficial role of antioxidants in the treatment of nanoparticle-induced toxicities. Oxid Med Cell Longev 2021:7244677 34820054 10.1155/2021/7244677 PMC8608524

[CR39] Molaei E, Molaei A, Abedi F, Hayes AW, Karimi G (2021) Nephroprotective activity of natural products against chemical toxicants: the role of Nrf2/ARE signaling pathway. Food Sci Nutr 9:3362–3384 34136201 10.1002/fsn3.2320 PMC8194945

[CR40] Møller P and Roursgaard M (2024) Gastrointestinal tract exposure to particles and DNA damage in animals: a review of studies before, during and after the peak of nanotoxicology. Mutat Res/Rev Mutat Res 108491 10.1016/j.mrrev.2024.108491 38522822

[CR41] Morgan A, Galal MK, Ogaly HA, Ibrahim MA, Abd-Elsalam RM, Noshy P (2017) Tiron ameliorates oxidative stress and inflammation in titanium dioxide nanoparticles induced nephrotoxicity of male rats. Biomed Pharmacother 93:779–787 28709131 10.1016/j.biopha.2017.07.006

[CR42] Mostafa HE-S, Alaa El-Din EA, El-Shafei DA, Abouhashem NS, Abouhashem AA (2021) Protective roles of thymoquinone and vildagliptin in manganese-induced nephrotoxicity in adult albino rats. Environ Sci Pollut Res 28:31174–31184 10.1007/s11356-021-12997-5 33595798

[CR43] Ohkawa H (1978) Assay for lipid peroxides in animal tissues by thiobarbituric acid reaction. Anal Biochem 98:351 10.1016/0003-2697(79)90738-3 36810

[CR44] Papp A, Horváth T, Igaz N, Gopisetty MK, Kiricsi M, Berkesi DS, Kozma G, Kónya Z, Wilhelm I and Patai R (2020) Presence of titanium and toxic effects observed in rat lungs, kidneys, and central nervous system in vivo and in cultured astrocytes in vitro on exposure by titanium dioxide nanorods. Int J Nanomed 9939–9960 10.2147/IJN.S275937 PMC7765755 33376320

[CR45] Parashar S, Raj S, Srivastava P, Singh AK (2024) Comparative toxicity assessment of selected nanoparticles using different experimental model organisms. J Pharmacol Toxicol Methods 130:107563 39357804 10.1016/j.vascn.2024.107563

[CR46] Peters RJ, Oomen AG, van Bemmel G, van Vliet L, Undas AK, Munniks S, Bleys RL, Tromp PC, Brand W, van der Lee M (2020) Silicon dioxide and titanium dioxide particles found in human tissues. Nanotoxicology 14:420–432 31994971 10.1080/17435390.2020.1718232

[CR47] Riaz M, Khalid R, Afzal M, Anjum F, Fatima H, Zia S, Rasool G, Egbuna C, Mtewa AG, Uche CZ (2023) Phytobioactive compounds as therapeutic agents for human diseases: a review. Food Sci Nutr 11:2500–2529 37324906 10.1002/fsn3.3308 PMC10261751

[CR48] Robb EL, Winkelmolen L, Visanji N, Brotchie J, Stuart JA (2008) Dietary resveratrol administration increases MnSOD expression and activity in mouse brain. Biochem Biophys Res Commun 372:254–259 18486604 10.1016/j.bbrc.2008.05.028

[CR49] Ryu AR, Bang IC, Lee SA, Lee MY (2016) The protective role of phytochemicals on TiO₂ nanoparticles-induced DNA damage in lymphocytes. J Environ Biol 37:913–917 29251483

[CR50] Shakibaei M, Harikumar KB, Aggarwal BB (2009) Resveratrol addiction: to die or not to die. Mol Nutr Food Res 53:115–128 19072742 10.1002/mnfr.200800148

[CR51] Shirdare M, Jabbari F, Salehzadeh M, Ziamajidi N, Nourian A, Heidarisasan S, Ghavimishamekh A, Taheri Azandariani M, Abbasalipourkabir R (2022) Curcuma reduces kidney and liver damage induced by titanium dioxide nanoparticles in male Wistar rats. Avicenna J Phytomed 12:537–547 36249452 10.22038/AJP.2021.53346.2727 PMC9516405

[CR52] Shukla RK, Kumar A, Vallabani NV, Pandey AK, Dhawan A (2014) Titanium dioxide nanoparticle-induced oxidative stress triggers DNA damage and hepatic injury in mice. Nanomedicine (Lond) 9:1423–1434 24367968 10.2217/nnm.13.100

[CR53] Son Y, Chung HT, Pae HO (2014) Differential effects of resveratrol and its natural analogs, piceatannol and 3,5,4′-trans-trimethoxystilbene, on anti-inflammatory heme oxigenase-1 expression in RAW264.7 macrophages. BioFactors 40:138–145 23861314 10.1002/biof.1108

[CR54] Struck MB, Andrutis KA, Ramirez HE, Battles AH (2011) Effect of a short-term fast on ketamine–xylazine anesthesia in rats. J Am Assoc Lab Anim Sci 50:344–348 21640029 PMC3103284

[CR55] Sun Y, Oberley LW, Li Y (1988) A simple method for clinical assay of superoxide dismutase. Clin Chem 34:497–500 3349599

[CR56] Sun Q, Tan D, Ze Y, Sang X, Liu X, Gui S, Cheng Z, Cheng J, Hu R, Gao G, Liu G, Zhu M, Zhao X, Sheng L, Wang L, Tang M, Hong F (2012) Pulmotoxicological effects caused by long-term titanium dioxide nanoparticles exposure in mice. J Hazard Mater 235–236:47–53 22898172 10.1016/j.jhazmat.2012.05.072

[CR57] Valentini X, Rugira P, Frau A, Tagliatti V, Conotte R, Laurent S, Colet J-M, Nonclercq D (2019) Hepatic and renal toxicity induced by TiO2 nanoparticles in rats: a morphological and metabonomic study. Journal of Toxicology 2019:5767012 30941172 10.1155/2019/5767012 PMC6421043

[CR58] Wang Y, Chen Z, Ba T, Pu J, Chen T, Song Y, Gu Y, Qian Q, Xu Y, Xiang K (2013) Susceptibility of young and adult rats to the oral toxicity of titanium dioxide nanoparticles. Small 9:1742–1752 22945798 10.1002/smll.201201185

[CR59] West JD, Ji C, Duncan ST, Amarnath V, Schneider C, Rizzo CJ, Brash AR, Marnett LJ (2004) Induction of apoptosis in colorectal carcinoma cells treated with 4-hydroxy-2-nonenal and structurally related aldehydic products of lipid peroxidation. Chem Res Toxicol 17:453–462 15089087 10.1021/tx034248o

[CR60] Xiao Z, Zheng M, Deng J, Shi Y, Jia M, Li W (2024) Nano-TiO2 regulates the MAPK (ERK, P38) pathway to promote apoptosis and inhibit proliferation of human colon cells. Ecotoxicol Environ Saf 283:116973 39213753 10.1016/j.ecoenv.2024.116973

[CR61] Yalcın T, Kaya S, Kuloğlu T (2024) Resveratrol may dose-dependently modulate nephrin and OTULIN levels in a doxorubicin-induced nephrotoxicity model. Toxicol Mech Methods 34:98–108 37807854 10.1080/15376516.2023.2268717

[CR62] Yang C, Zhu Y, Guan C, Wang L, Xu L, Li D, Zhang S, Zhang L, Yang D, Xu Y (2021) Crystal phase-related toxicity of one-dimensional titanium dioxide nanomaterials on kidney cells. ACS Appl Bio Mater 4:3499–3506 35014434 10.1021/acsabm.1c00036

[CR63] Yörükoğlu K (2021) Nükleer ve Sitoplazmik Boyama: Rutin Hematoksilen+ Eosin. Turkiye Klinikleri Med Patholy-Spec Top 6:61-65

